# Editorial: Public health and health research data: availability, needs and challenges

**DOI:** 10.3389/fpubh.2023.1340663

**Published:** 2023-12-15

**Authors:** Paulo Jorge Nogueira, Andreia Silva Costa, Carla Sofia e Sá Farinha

**Affiliations:** ^1^National School of Public Health, New University of Lisbon, Lisbon, Portugal; ^2^Comprehensive Health Research Center, New University of Lisbon, Lisbon, Portugal; ^3^Institute of Environmental Health, Faculty of Medicine, University of Lisbon, Lisbon, Portugal; ^4^Centro de Investigação, Inovação e Desenvolvimento em Enfermagem de Lisboa, escola Superior de Enfermagem de Lisboa, Lisbon, Portugal; ^5^Center for Environmental and Sustainability Research, Faculty of Sciences and Technology, New University of Lisbon, Caparica, Portugal

**Keywords:** public health data, COVID-19 pandemic, healthcare policy, data analysis in healthcare, cyberinfrastructure in health, global health strategy

## The criticality of data in the COVID-19 era

In the era of COVID-19, the availability and quality of public health data have become more critical than ever. This pandemic has highlighted the essential role of reliable data in informing health policy and public health management. The works of Ortiz-Prado et al. (1) and Zhang et al. underscore the importance of accessible and high-quality health data in formulating effective health strategies, especially during crises. Johannesson et al. emphasize the need for comprehensive and coordinated data collection and sharing systems, transcending national boundaries to tackle global health challenges effectively.

## Integrating data for comprehensive health overview

The pandemic has illuminated significant challenges in managing health emergencies, particularly in resource-limited settings. Naz et al. stress the importance of integrating data from multiple sources, including governmental and private sectors, to create a comprehensive health overview, which is a necessity in low- and middle-income countries (LMICs). The study by Gao et al. demonstrates how cyberinfrastructure can augment traditional medical infrastructure, supporting adaptable policies and responses to public health emergencies.

## Case studies and insights

- Ortiz-Prado et al. (1) advocate for measures that foster freedom of expression and sharing of scientific knowledge, which are vital for the growth and development of countries.
- Zhang et al.. highlight the necessity of cooperation to increase access to essential medications, contributing to universal health care goals.
- Ouedraogo et al. shows that despite non-communicable diseases (NCDs) being a major cause of mortality, they are often underfunded in health budgets.
- Lyons and Bhagwandeen discuss how access and availability of public healthcare services affect migrants and refugees during public health emergencies.
- Lv et al. and Gavurova et al. focus on using data for improved decision-making and developing tools for health-related project selection processes.
- The Role of Data in Public Health Management.
- The UNITE Summit 2023 Health Care Think Tank symbolizes the growing engagement in transforming health data management. This initiative aims to define key initiatives to tackle major challenges in health data management.
- Wang et al. provide an analysis of the global, regional, and national burden of digestive diseases, providing a critical perspective for public health initiatives based on the Global Burden of Disease Study 2019.
- Abdulla et al. give insights into the intersection of infectious diseases and nutritional challenges, highlighting food insecurity among tuberculosis patients in Eastern Ethiopia.
- Mutale et al. evaluate a protocol-driven primary care model in rural Zambia aimed at reducing all-cause mortality, emphasizing integrated healthcare approaches.
- Junker et al. propose the development of a high-frequency mental health surveillance prototype in Germany, underlining the importance of data infrastructure in public health.
- Panag et al. perform a comparative analysis of national surveillance reporting for Mpox virus in various countries, stressing the need for standardized reporting in global public health.
- and, Shen and Wang discuss regulating China's health code system for future pandemic preparedness, reflecting on digital health strategies and public health policy.

## Conclusion: harnessing the true power of data in public health

This Research Topic, “Public Health and Health Research Data: Availability Needs and Challenges,” has demonstrated unequivocally that the true power in data lies not merely in its existence, but in its strategic availability, analysis, and application in public health decision-making. The COVID-19 pandemic has served as a critical catalyst, underscoring this reality and emphasizing the urgent need for robust, accessible health data systems.

Throughout this Research Topic, studies such as those by Ortiz-Prado et al. (1), Zhang et al., and others, have provided compelling evidence of how data availability and quality directly impact the effectiveness of health strategies and responses, particularly in crisis situations. The case studies highlighted reveal that comprehensive, coordinated data collection and sharing systems are not just beneficial but essential for addressing global health challenges.

We have seen that in resource-limited settings, the integration of data from diverse sources, including governmental and private sectors, is crucial for creating a comprehensive health overview, as emphasized by Naz et al.. Additionally, the role of cyberinfrastructure, as shown by Gao et al., in augmenting traditional healthcare systems, presents new avenues for public health management and resilience against future emergencies.

As we move forward, it is imperative to foster collaboration across sectors and embrace a One Health perspective that recognizes the interconnectedness of human, animal, and environmental health. This comprehensive approach is vital for refining health data systems and effectively responding to current and future public health challenges.

In conclusion, this Research Topic has not only highlighted the current shortfalls in health data management but also pointed toward potential solutions and innovative technological pathways. The insights gained here advocate for a more inclusive approach to data gathering and utilization at all levels—governmental, private, and grassroots. The lesson is clear: the true power of data in public health emerges when it is made available, analyzed thoroughly, and utilized strategically to inform and guide public health policies and interventions. The future of public health lies in harnessing the full potential of data, ensuring that it becomes a cornerstone of public health decision-making and policy development in our increasingly interconnected world.

## Author contributions

PN: Writing – original draft, Writing – review & editing. AC: Writing – review & editing. CF: Writing – original draft, Writing – review & editing.
